# Frequencies Evaluation of β-Casein Gene Polymorphisms in Dairy Cows Reared in Central Italy

**DOI:** 10.3390/ani10020252

**Published:** 2020-02-05

**Authors:** Carla Sebastiani, Chiara Arcangeli, Marcella Ciullo, Martina Torricelli, Giulia Cinti, Stefano Fisichella, Massimo Biagetti

**Affiliations:** 1 Istituto Zooprofilattico Sperimentale dell’Umbria e delle Marche-Togo Rosati (IZSUM), Via Salvemini 1, 06126 Perugia, Italy; c.arcangeli@izsum.it (C.A.); m.ciullo@izsum.it (M.C.); m.torricelli@izsum.it (M.T.); s.fisichella@izsum.it (S.F.); 2 R&D Cooperlat, Società Cooperativa Agricola, via Piandelmedico 74, 60035 Jesi (Ancona), Italy; g.cinti@trevalli.cooperlat.it

**Keywords:** β-casein, polymorphisms, bovine, milk

## Abstract

**Simple Summary:**

Bovine milk contains several β-casein variants, with the A1 and A2 variants occurring most frequently. The presence of some variants, such as A1, B, and C, is considered a risk factor for disease in humans who consume milk. These variants are probably involved in intolerance to milk and some human diseases due to the production of a bioactive peptide with opioid activity during digestion, β-casomorphin 7 (BCM-7). In contrast, the A2 variant is not involved in pathogenetic mechanisms; thus, its presence in milk is a desirable feature. The difference between the A1 and A2 variants is a mutation at position 67 of the β-casein gene (*CSN2*), which causes an amino acid to change from histidine (in the A1, B, and C variants) to proline (in the A2 variant). To select dairy cows on the basis of the presence of the β-casein variant A2, allele frequencies of *CSN2* variants were evaluated in Italian dairy cows reared in central Italy. The results of this study may help with the selection of animals with the β-casein gene variant A2 to produce a more digestible milk that only contains the β-casein variant A2.

**Abstract:**

The majority of proteins in cow’s milk are caseins, which occur in four groups (α-s1, α-s2, β, and k) encoded by different genes (*CSN1S1*, *CSN1S2*, *CSN2*, and *CSN3*, respectively). In this study, we focused on the β-casein allele variants A1 and A2 due to their influence on milk’s technological characteristics and human health. Digestion of the β-casein variant A1 leads to the formation of β-casomorphin 7 (BCM-7), a bioactive peptide that has been suggested to be a possible cause of various human diseases and associated with low milk digestibility. The potential negative role of the β-casein variant A1 in human health has stimulated the planning of cattle breeding programs based on genetic selection to increase the frequency of the A2 variant, which is associated with increased milk digestibility. The aim of this work was to evaluate the frequencies of the different β-casein variants in Italian Holstein Friesian dairy cows from cattle farms located in central Italy to select a population of A2 homozygous animals. β-casein genotypes were identified by evaluating the presence of single nucleotide polymorphisms (SNPs) of the *CSN2* gene using PCR and sequencing analysis. The frequency of the desirable β-casein variant A2 in the studied bovine population was 0.61. The frequency of the undesirable A1 variant in the studied bovine population was 0.30. The frequency of the A2 allele was higher than expected for the breed; therefore, genetic selection for the A2 variant in these animals could be achieved in a fairly short time using A2 homozygous bulls.

## 1. Introduction

Cow’s milk is considered an important source of food for humans and plays a fundamental role in human health due to its valuable protein, fat-soluble vitamins, and mineral salt content, particularly calcium, which counteracts bone fragility and the risk of osteoporosis. Bovine milk is composed of 87% water, 3.68% lipids, 3.51% proteins, 4.98% lactose, and 0.74% microelements [1]. Of the proteins, the most important are caseins, which represent 82% of the total protein content [2]. Caseins are subdivided into four groups, αs1, αs2, β, and k, respectively encoded by the *CSN1S1*, *CSN1S2*, *CSN2*, and *CSN3* genes located on chromosome 6 [3]. The main function of caseins is the transport of calcium phosphate [4]. However, β-casein, which accounts for 36% of the total protein content, is also important for curd formation and determining the surface properties of micelles, which are useful features for cheese production [5,6]. Dairy cattle have 12 β-casein variants (A1, A2, A3, B, C, D, E, F, H1, H2, I, and G); however, only seven of these (A1, A2, A3, B, C, I, and E) have been detected in European cattle breeds (Table 1) [7,8]. The A2 variant is considered the oldest variant, from which the others originated via mutation. The most common variants are A1 and A2; the B variant is less common [9]. The I variant is usually detected at low frequencies [8], the A3 and C variants are rare [9], the E variant is detectable only in the Italian Piedmontese breed [10], and the F variant has been detected in the Emilia Romagna region (Northern Italy) at a very low frequency (0.006) [8]. The difference between the A1 and A2 variants is due to a mutation in position 67, which causes an amino acid to change from histidine (in the A1, B, and C variants) to proline (in the A2, A3, E, and I variants). The allele variants A1, B, and C differ from each other with respect to an amino acid in position 122 (serine in A1 and C, arginine in B) and one in position 37 (glutamic acid in the A1 and B variants and lysine in the C variant). The I and A3 variants were derived from a mutation of the A2 variant; specifically, the I variant has a leucine instead of a methionine in position 93 and the A3 variant has a glutamine instead of a histidine in position 106. To summarize, the A1 and A3 variants originated from the A2 variant; subsequently, the I variant was derived from the A3 variant, and the B and C variants were derived from the A1 variant. Each variant exhibits the same behavior as the variants from which it arose in terms of β-casomorphin 7 (BCM-7) formation [11] (Table 1).

We paid particular attention to the allele variants A1 and A2 due to their influence on milk’s technological characteristics and implications for human health. The A1 variant improves curd consistency, milk coagulation, and micelle size, but results in lower milk digestibility compared with the A2 variant [12]. The β-casein variants A1 and A2 are differently processed; digestive enzymes are able to perform a proteolytic cleavage of the β-casein chain when a histidine is present at position 67 to form a peptide of seven amino acids named β-casomorphine 7 (BCM-7). Evidence showed that BCM-7 has strong opioid activity with an additional oxidizing effect and can interact with endogenous opioid systems at the gastrointestinal wall in both newborns and adults [13]. According to Deth et al. (14), consumption of milk containing the A2 variant increases the natural production of glutathione (GSH), an antioxidant that is widely recognized for its association with a series of health benefits. The study showed that the consumption of milk containing only the A2 variant increases the GSH level in the blood to approximately twice the level associated with the consumption of milk containing both the β-casein variants A1 and A2 [14]. Therefore, the allele variants that are considered risk factors for human disease are those that contain a histidine in position 67 (A1, B, and C variants), whereas those that contain a proline, i.e., the A3 and I variants, behave like the A2 variant even though they contain other single-nucleotide polymorphisms (SNPs) in position 106 (histidine to glutamine) and position 93 (methionine to leucine), respectively. Some studies reported a correlation between the consumption of milk containing the β-casein variant A1 and heart disease, sudden infant death syndrome, and aggravation of symptoms associated with schizophrenia, autism, type 1 diabetes, and milk intolerance [15,16,17,18,19,20].

In contrast, a scientific report of the European Food Safety Authority (EFSA) in 2009 concluded that no cause–effect relationship exists between the consumption of milk containing the A1 variant and the etiology of the abovementioned diseases and that further investigations are necessary [13]. More recently, a scientific report highlighted a lack of a correlation between adverse human health effects and consumption of the β-casein variant A1 in comparison to the β-casein variant A2 [21].

Therefore, the discussion on the adverse human health effects of the β-casein variant A1 remains open. However, milk obtained from cows with the A2/A2 genotype seems to be more digestible than milk obtained from cows with the A1 genotype in terms of an increase in gastrointestinal transit [12]. A2 cow’s milk, or milk without the A1 variant, is commercially available in a number of countries, including Australia, the United Kingdom, the United States, New Zealand, and the Netherlands, and is widely recommended for people who are milk-intolerant. Formula for newborns that contains the β-casein variant A2 is now sold in China and Australia and is promoted commercially as being more gentle on an infant’s digestive system [12]. The potential negative role of the β-casein variant A1 in human health has stimulated the planning of cattle breeding programs based on the selection of β-casein gene polymorphisms.

The purpose of this work was to evaluate the frequency of occurrence of different β-casein variants in dairy cows from cattle farms in central Italy that supply milk to an important drinking-milk-producing plant, with the aim of selecting a population of A2 homozygous animals and commercializing an A2 milk that only contains the β-casein variant A2.

## 2. Materials and Methods

A total of 1629 whole blood samples were collected from Italian Holstein Friesian cows from 17 farms located in central Italy. The samples were collected in tubes containing Ethylenediaminetetra-acetic acid (EDTA) as an anticoagulant and stored at −20 °C until analysis. The samples were taken in a single withdrawal, at the same time of the mandatory periodic tests required by Italian National Health Programs and during farmer’s voluntary health controls. Genomic DNA was extracted using a High Pure PCR Template Preparation Kit (Roche Life Science, Mannheim, Germany) according to the manufacturer’s instructions. Specific primers for the exon 6 and 7 portions of the *CSN2* gene were selected from the literature [22] and modified using Primer Express® v3.0.1 software (Applied Biosystems; Thermo Fisher Scientific Inc., Waltham, MA, USA) to increase the efficiency of the assay (Table 2).

The PCR was set up in a total reaction volume of 50 µL as follows: for exon 6, 1.5 X GoTaq® Flexi Buffer, 1 mM MgCl_2_, 2.5 U of GoTaq® G2 Flexi DNA Polymerase (Promega Corporation, Madison, WI, USA), 200 µM dNTPs (GE Healthcare, Buckinghamshire, England), 0.3 µM of each primer (Invitrogen; Thermo Fisher Scientific Inc., Waltham, MA, USA) (Table 2), and 4 µL of the extracted DNA template; for exon 7, 1 X GoTaq® Flexi Buffer, 1.5 mM MgCl_2_, 2.5 U of GoTaq® G2 Flexi DNA Polymerase (Promega Corporation) 200 µM dNTPs (GE Healthcare), 0.2 µM of each primer (Invitrogen; Thermo Fisher Scientific Inc.) (Table 2) and 2 µL of the extracted DNA template.

PCR amplifications for both protocols were conducted on a Mastercycler Ep Gradient S (Eppendorf AG, Hamburg, Germany) with the following thermal cycling profile: an initial denaturation at 95 °C for 10 min, followed by 35 cycles at 95 °C for 1 min, 58 °C for 1 min, 72 °C for 1 min, and a final elongation step at 72 °C for 7 min. Amplification products were analyzed by electrophoresis on 2% agarose gel containing Midori Green Advanced DNA Stain (Nippon Genetics Europe GmbH, Düren, Germany) and purified with a QIAquick® PCR Purification Kit (Qiagen, Hilden, Germany), according to the manufacturer’s instructions. Sequencing reactions were completed for both strands using a BrilliantDye^TM^ Terminator Cycle Sequencing Kit v3.1 (NimaGen BV, Nijmegen, The Netherlands) according to the manufacturer’s instructions with the same primers that were used for PCR amplification. Sequencing reactions were analyzed in a 3500 Genetic Analyzer (Applied Biosystems; Thermo Fisher Scientific Inc.). Sequences were then aligned to the bovine β-casein gene (Accession number X14711.1) with the ClustalW tool of the BioEdit v7.2.5 software [23]. Electropherograms were checked at each investigated mutation point to identify heterozygous peaks indicating the presence of both alleles. The polymorphisms at positions 36 and 37 of exon 6 and positions 67, 72, 88, 93, 106, 122, and 138 of exon 7 were analyzed to discriminate between the different β-casein variants.

Allele and genotype frequencies were directly calculated dividing the number of copies of each allele and genotype by the total alleles and by the total individuals, respectively. The Hardy-Weinberg (HW) equilibrium was verified using Chi-square test (*p* < 0.05) by Genalex 6.5 software [24,25].

## 3. Results and Discussion

The main goal of our study was to evaluate the frequency of occurrence of the β-casein *CSN2* gene alleles in dairy cattle of the Italian Holstein Friesian breed reared in central Italy. We paid particular attention to the frequencies of the A2 variant and its related genotypes given its association with health benefits. Although the Italian Holstein Friesian breed is not among those breeds characterized by the highest frequency of the A2/A2 genotype, it has a sufficiently high frequency to allow for effective genetic selection of this feature [26]. Therefore, the estimation of allele and genotype frequencies is important for planning an efficacious genetic selection program for these animals with the final goal of producing A2 cow’s milk. Our results show the distribution of the allele and genotype frequencies of the different *CSN2* gene variants in the population under study. Considering the polymorphic sites evaluated in this study, all the alleles conformed to the HW equilibrium and no deviation was detected.

The sequencing analysis performed in both directions on the PCR products showed that, of the nine considered polymorphic sites spanning the regions of exons 6 and 7 of the *CSN2* gene, a total of five were polymorphic: E37K, P67H, M93L, H106Q, and S122R (Table 1). They produced six β-casein variants (A1, A2, A3, B, C, and I) in 13 genotypes, 3 of which were homozygous (A1/A1, A2/A2, and B/B) and 10 of which were heterozygous (A1/A2, A1/A3, A1/B, A1/C, A1/I, A2/A3, A2/B, A2/I, A3/B, and B/I) as shown in Table 3. 

The A2 allele was the most commonly found, with a frequency of 60.65%, followed by the A1 allele with a frequency of 30.39%, the B allele at 5.68%, the I allele at 3.10%, the A3 allele at 0.15%, and the C allele with a frequency of 0.03%. These results agree with those reported from a study conducted in cattle of the same breed from farms located in the Emilia Romagna region (Northern Italy) by Massella et al. (8), who found a high frequency of the A2 allele (54.6%), followed by the A1 (37.1%), B (5.0%), I (2.7%), and F (0.6%) alleles.

In our samples, we did not observe the F variant, which is very rare and for which few data are available about its frequency in milk-producing breeds [8]. We did not find the E variant, which, to date, has only been observed in the Piemontese breed [27] or the D, G, H1, and H2 variants, which have never been found in European breeds [7]. These results are also consistent with those reported by Kaminsky et al. (16), which showed that the A1 and A2 variants are the most common in the Holstein Friesian breed.

Regarding the genotype, the homozygous genotype A2/A2 was the most common, with a frequency of 36.96%, followed by the heterozygous genotypes A1/A2 and A1/A1 with frequencies of 35.79% and 9.88%, respectively. The remaining genotypes showed lower frequencies that ranged from 7.55% (A2/B) to 0.06% (A1/A3–A1/C) (Table 3). A total of 2.46% of the examined animals were found to carry the A3 and I variants (A1/I, B/I, A3/B, and A1/A3), while 4% were A2/A3 and A2/I. These individuals could be considered as A2 heterozygous and homozygous, respectively.

A breed is considered suitable for selection of the A2 allele if its frequency is close to 50%. In this case, given the Mendelian inheritance of the trait and presuming that the population is in Hardy-Weinberg equilibrium, in the herd we would expect to find 25% homozygous A1/A1 animals, 50% A1/A2 heterozygotes, and 25% A2/A2 homozygous individuals, and that the produced bulk milk contains 50% β-casein variant A1 and 50% β-casein variant A2 [26]. To change the herd’s composition with the aim of producing a milk containing only the β-casein variant A2, A2/A2 bulls must be used for the artificial insemination of A2 carrier cows. The Italian Holstein Friesian breed provides an appropriate genetic background for increasing the frequency of this favorable allele through appropriate breeding.

In the farms analyzed, we found an A2 allele frequency of 60.65%, slightly greater than those reported in the recent literature (8). The presence of other variants similar to A2 (A3 and I), relative to BMC-7 production, accounting for another 3.25%, increased the frequency of favorable variants to 64%. Consequently, we found high frequencies of A2/A2 individuals (37%) and A2 heterozygous carriers (47.4%). As the A3 and I variants behave similarly to the A2 variant, the percentage of A2/A2-like genotypes (A2/A2, A2/A3, and A2/I) was 41%, increasing the overall percentage of genotypes with at least one A2-like allele to 87%.

## 4. Conclusions

In this study, we focused on the β-casein variants A1 and A2 due to their influence on milk’s technological characteristics and their implications for human health. The β-casein variant A2 is desirable in milk because it increases the digestibility of milk. A2 cow’s milk can be immediately produced in a herd if the cows present on farms are screened to identify animals with the A2/A2 genotype and the milk of these individuals is separated from that of the others. The identification of A2 carrier cows then allows the planning of animal couplings with the aim of generating A2 homozygous progenies. In conclusion, genotyping by sequencing is a fast and reliable method for monitoring the allele frequencies of β-casein variants in a population of dairy cows where selection of the β-casein variant A2 is the aim.

Marker-assisted selection (MAS) should be applied to align Italian dairy breeds with those of other countries that have already invested in A2 cow’s milk production. In many countries, A2 cow’s milk has already been commercialized as a product with beneficial properties with a resulting economic gain.

These data will allow us to manage animal couplings to increase the frequency of the favorable A2 allele, simultaneously decrease the frequency of undesirable alleles, and finally create herds of A2/A2 animals from the population of dairy cow breeds oriented to A2 cow’s milk production.

## Figures and Tables

**Table 1:** The change in the amino acid sequence of β-casein variants (in bold: amino acid variations).

βcasein Variant	Amino Acid Position								
	36	37	67	72	88	93	106	122	138
A2 *	Glu (E)	Glu (E)	Pro (P)	Gln (Q)	Leu (L)	Met (M)	His (H)	Ser (S)	Pro (P)
A1 *	Glu (E)	Glu (E)	His (H)	Gln (Q)	Leu (L)	Met (M)	His (H)	Ser (S)	Pro (P)
A3 *	Glu (E)	Glu (E)	Pro (P)	Gln (Q)	Leu (L)	Met (M)	Gln (Q)	Ser (S)	Pro (P)
B *	Glu (E)	Glu (E)	His (H)	Gln (Q)	Leu (L)	Met (M)	His (H)	Arg (R)	Pro (P)
C *	Glu (E)	Lys (K)	His (H)	Gln (Q)	Leu (L)	Met (M)	His (H)	Ser (S)	Pro (P)
E *	Lys (K)	Glu (E)	Pro (P)	Gln (Q)	Leu (L)	Met (M)	His (H)	Ser (S)	Pro (P)
I *	Glu (E)	Glu (E)	Pro (P)	Gln (Q)	Leu (L)	Leu (L)	His (H)	Ser (S)	Pro (P)
D	Glu (E)	Glu (E)	Pro (P)	Gln (Q)	Leu (L)	Met (M)	His (H)	Ser (S)	Pro (P)
F	Glu (E)	Glu (E)	His (H)	Gln (Q)	Leu (L)	Met (M)	His (H)	Ser (S)	Leu (L)
G	Glu (E)	Glu (E)	His (H)	Gln (Q)	Leu (L)	Met (M)	His (H)	Leu (L)	Pro (P)
H1	Glu (E)	Glu (E)	Pro (P)	Gln (Q)	Ile (I)	Met (M)	His (H)	Ser (S)	Pro (P)
H2	Glu (E)	Glu (E)	Pro (P)	Glu (E)	Leu (L)	Leu (L)	His (H)	Ser (S)	Glu (E)

Arg: arginine; Gln: glutamine; Glu: glutamic acid; His: histidine; Ile: isoleucine; Leu: leucine; Lys: lysine; Met: methionine; Pro: proline; Ser: serine; * Allele variants detected in European cattle breeds.

**Table 2:** Primer sequences (For, forward primer; Rev, reverse primer).

Target Gene	Target Sequence	Primer Sequences	Amplification Product (bp)	Reference
*CSN2*	Exon 6	For CATCAATAAGGTAAAACCCCTCATATT Rev TTGTCAAAGTTTTTATTTCTTGCACTG	274	This study
	Exon 7	For TTTCCAGGATGAACTCCAGGAT Rev CATCAGAAGTTAAACAGGCACAGTTAG	547	

**Table 3:** The allele and genotype frequencies (%) in the examined animals (the data are sorted by decreasing allele and genotype frequency).

Allele	Allele Frequency (%)	Genotype	Genotype Frequency (%)
A2	60.65	A2/A2	36.96
A1	30.39	A1/A2	35.79
B	5.68	A1/A1	9.88
I	3.10	A2/B	7.55
A3	0.15	A2/I	3.93
C	0.03	A1/B	3.07
		A1/I	2.03
		B/I	0.25
		B/B	0.18
		A2/A3	0.12
		A3/B	0.12
		A1/A3	0.06
		A1/C	0.06
